# Antibacterial Activity and Proposed Mode of Action of Extracts from Selected Zimbabwean Medicinal Plants against *Acinetobacter baumannii*

**DOI:** 10.1155/2024/8858665

**Published:** 2024-08-23

**Authors:** Auxillia Machingauta, Stanley Mukanganyama

**Affiliations:** ^1^ Bio-Molecular Interactions Analyses Group Department of Biotechnology and Biochemistry University of Zimbabwe, Mt Pleasant, P.O. Box 167, Harare, Zimbabwe; ^2^ Department of Therapeutics Natural Products Research Unit African Institute of Biomedical Science and Technology Wilkins Hospital, Block C, Corner J. Tongogara and R. Tangwena, Harare, Zimbabwe

## Abstract

*Acinetobacter baumannii* was identified by the WHO as a priority pathogen in which the research and development of new antibiotics is urgently needed. Plant phytochemicals have potential as sources of new antimicrobials. The objective of the study was to determine the antibacterial activity of extracts of selected Zimbabwean medicinal plants against *A. baumannii* and determine their possible mode of action. Extracts were prepared from the leaves of the eight plants including the bark of *Erythrina abyssinica* using solvents of different polarities. Antibacterial activity was evaluated using the microbroth dilution method coupled with the *in vitro* iodonitrotetrazolium colorimetric assay. The effect of the extracts on membrane integrity was determined by quantifying the amount of protein and nucleic acid leaked from the cells after exposure to the extracts. The effects of the extracts on biofilms were investigated. Toxicity studies were carried out using sheep erythrocytes and murine peritoneal cells. Seven out of eight evaluated plant extracts were found to have antibacterial activity. The *Combretum apiculatum* acetonie (CAA) extract showed the highest inhibitory activity against *A. baumannii* with a minimal inhibitory concentration of 125 *µ*g/mL. The minimum inhibitory concentration (MIC) of the CAA extract caused a protein leakage of 32 *µ*g/mL from *A. baumannii*. The *Combretum apiculatum* acetonie (CAA), *C. apiculatum* methanolic (CAM), *Combretum zeyheri* methanolic (CZM), and *Erythrina abyssinica* methanolic (EAM) extracts inhibited *A. baumannii* biofilm formation. The EAM extract was shown to disrupt mature biofilms. The potent extracts were nontoxic to sheep erythrocytes and mouse peritoneal cells. The activities shown by the extracts indicate that the plants have potential as sources of effective antibacterial and antibiofilm formation agents against *A. baumannii*.

## 1. Introduction

Antimicrobial resistance is one of the most serious public health threats of the twenty-first century [1]. ESKAPE pathogens (*Enterococcus faecium, Staphylococcus aureus, Klebsiella pneumoniae, Acinetobacter baumannii, Pseudomonas aeruginosa*, and *Enterobacter* species) are a group of six pathogens with increasing resistance to the available antibacterial agents and virulence in humans [2]. The group is known to effectively evade the biocidal action of antimicrobial agents and is responsible for the majority of nosocomial infections [2]. The emergence and spread of multidrug-resistant (MDR) bacterial pathogens have led to increased bacterial resistance to most antibiotics used in therapy [3]. In a study conducted in Zimbabwe, an increased emergence of carbapenem-resistant *A. baumannii* has been observed [4].

The World Health Organization (WHO) declared carbapenem-resistant *A. baumannii* a priority pathogen in which studies and the development of new antibiotics are urgently needed [5]. *A. baumannii* is a strictly aerobic Gram-negative coccobacillus [6]. The pathogen is an opportunist that takes advantage of patients with a compromised immune system, causing a variety of infections [7]. The most common infections with *A. baumannii* include ventilator-associated pneumonia, meningitis, wound and soft tissue infections, and urinary tract infections [8]. The challenges involved in the treatment of *A. baumannii* infections are mainly due to intrinsic and acquired resistance to multiple classes of antibiotics [9]. The mechanisms of resistance of *A. baumannii* include enzymatic degradation of drugs, target site modification, multidrug efflux pumps, and membrane permeability defects [10, 11].

Medicinal plants are an important source of therapeutics and a source of potential new medicines [12]. Phytomedicines play a major role in the traditional system of healing, especially in developing countries [13]. According to WHO, 80% of the world's population is dependent on traditional therapies [14]. Plants contribute to a variety of chemical compounds called phytochemicals. These phytochemicals have therapeutic properties [15]. Scientific studies to determine the therapeutic potential of phytochemicals from plants are important for the development of novel antibiotics [16]. Zimbabwean plants have been shown to have antibacterial, antifungal, and antiproliferative effects [17]. These include plants from the *Combretum* species, *Triumfetta welwitschii, Parinari curatellifolia, Vernonia adoensis*, and *Myricacea* species [17, 18]. Information on the medicinal plants used in this study is shown in Table 1. The main objective of this study was to determine the antibacterial activity of eight plant extracts from selected medicinal plants from Zimbabwe against a priority pathogen, *A. baumannii*. In addition, an evaluation of the possible mode of action of the most potent extracts was also carried out.

## 2. Materials and Methods

### 2.1. Plant Collection and Authentication

The medicinal plants used in this study were collected from the Centenary communal lands (geographical coordinates 16.7294°S, 31.1166°E; 1213.42 meters above sea level) in the Mashonaland Central Province of Zimbabwe, the University of Zimbabwe, Mt. Pleasant Harare Province of Zimbabwe (17.7824°S, 31.0546 E 1,483 meters above sea level), and Norton, Mashonaland Province of Zimbabwe (17.8765°S, 30.6742 E 1378.71 meters above sea level). Christopher Chapano, a taxonomist at the National Herbarium and Botanic Gardens (Harare, Zimbabwe), authenticated the plants. The plants used in this study were *Callistemon citrinus*, *Combretum apiculatum*, *Combretum zeyheri, Combretum molle, Combretum platypetalum, Erythrina abyssinica, Parinari curatellifolia,* and *Syzygium guineense*. The plants were collected from January 15 to January 31, 2021, during the summer period in Zimbabwe. Not all parts of the plant were used for conservation purposes. For plants used as herbal medicines in Zimbabwe, roots are used in approximately 60% of cases, followed by 35% for leaves [24]. Based on their ethnomedicinal use in Zimbabwe, as well as the need for sustainable harvesting and conservation, leaves were chosen to be used in this study.

Voucher samples (*Callistemon citrinus-*UZ2 E7, *Combretum apiculatum-*C1 E7, *Combretum zeyheri-*N6 E7, *Combretum molle*-C9 E7, *Combretum platypetalum*-N9 E7*, Erythrina abyssinica*-UZ11E7, *Parinari curatellifollia*-C6 E7, and *Syzygium guineense*-C12 E7) were deposited in the herbarium in the Department of Biotechnology and Biochemistry at the University of Zimbabwe.

### 2.2. Preparation of Plant Extracts

All the solvents used in this study were analytical grade and were obtained from Sigma-Aldrich (Darmstadt, Germany). The leaves, bark, or flowers of the plant were dried at 40°C in a hot air incubator (Labotec Co., Cape Town, South Africa). The dried plant parts were ground to a fine homogeneous powder using mortar and pestle. The cold maceration method was used to extract phytochemicals from powdered leaves, bark, or flowers. Maceration involved soaking plant materials with a suitable menstrual sample in a beaker covered with foil paper and separation of the micelle from the marc by filtration [25]. In total extraction, a 20 g mass of leaf powder was placed in a beaker and 100 mL of 50 : 50 v/v dichloromethane (DCM): methanol or water: ethanol was added to the beaker to ensure that the leaf powder was submerged. This procedure was carried out for all the leaves of the 8 plant species including the bark of *Erythrina abyssinica.*

Serial exhaustive extraction involves successive extraction with solvents of increasing polarity from hexane (nonpolar) to water (polar), ensuring the extraction of a wide range of phytochemicals with different polarities [26]. Serial extraction of a new powder sample was performed using hexane, DCM, acetone, ethyl acetate, methanol, ethanol, and water. The container was covered with foil paper and allowed to stand at room temperature overnight with frequent agitation. The mixture was filtered using Whatman filter paper no. 2 (Sigma Aldrich, Darmstadt, Germany). The filtrate was allowed to dry under a fan in the fume hood cabinet. To ensure that there were no solvent residues, the samples were also dried to dry powder using a 40°C oven for 24 hours before use. The dried extract was weighed and stored in sterile tubes at−4°C. In serial exhaustive extraction, the residue was retained and allowed to dry for further extraction using dichloromethane (DCM), acetone, ethyl acetate (EA), ethanol, methanol, and water. The percentage yield for the various extracts was calculated using the following formula:

(1) $$\begin{array}{c} \text{Percentage yield}\left(\%\right)=\frac{\text{Mass of extract obtained }\left(g\right)\times 100}{\text{Mass of plant powder used }\left(g\right)}. \end{array}$$

The serial exhaustive extraction was carried out for all the leaves of the 8 plant species including the bark of *Erythrina abyssinica.*

### 2.3. Chemical and Reagents

The chemicals used in this study include dimethyl sulfoxide (DMSO), ciprofloxacin, polymyxin B sulfate, (2–(4-iodophenyl)-3–(4-nitrophenyl)-5-phenyl-2H-tetrazolium chloride) INT, Coomassie Brilliant blue G-250, propidium iodide (PI), crystal violet, and sodium dodecyl sulfate (SDS). These were purchased from Sigma-Aldrich (Darmstadt, Germany). Tryptic soy broth (TSB), tryptic soy agar (TSA), and Muller–Hinton broth (MHB) were also purchased from Sigma-Aldrich (Darmstadt, Germany).

### 2.4. Microbial Strains and Culture Media

*A. baumannii,* a nosocomial pathogen on the critical priority list as stated by the World Health Organization [5] was chosen for the study. *A. baumannii* CECTTM (the University of Valencia) 9111 Vitroid^TM^ strain (ATCC® 19606) was purchased from Sigma-Aldrich (Darmstadt, Germany). The type strain ATCC 19606 has been widely used in studying the virulence, pathogenesis, and mechanisms of antimicrobial resistance in *A. baumannii*. The susceptibility and virulence profile of the strain used as a model in this study is given by Hamidan and Zhu [27, 28]. This strain is resistant to ampicillin, streptomycin, sulfamethoxazole, and chloramphenicol but is sensitive to colistin, polymycin B, ciprofloxacin, meropenem, tetracycline, rifampicin, and tobramycin [27, 28]. The bacteria were kept in glycerol stocks at −35°C. For each assay, bacteria were grown on tryptic soy agar for 24 hours at 37°C, followed by inoculation in Muller–Hinton broth. The cells were grown in Muller-Hinton broth overnight. Cell densities were standardised according to 0.5 McFarland standards to create inoculum densities of 2 × 10^6^ CFU/mL for use in the antibacterial assay.

### 2.5. Determination of Antibacterial Activities

DCM: methanol, water: ethanol, hexane, DCM, ethyl acetate, acetone, methanol, ethanol, and water extracts from 8 Zimbabwean medicinal plants were evaluated for antibacterial activity against *A. baumannii* using the broth microdilution method according to the European Committee on Antimicrobial Susceptibility Testing (EUCAST) protocol [29] and Eloff, [30] with some adjustments. The extracts were reconstituted in DMSO. Extract concentrations of 125, 250, 500, and 1000 *μ*g/mL were obtained by serially diluting these with Muller–Hinton broth. A volume of 100 *μ*L extract or polymyxin B was added separately to the wells of a 96-well microplate. A volume of 100 *μ*L of bacterial cells with a final concentration of 2 × 10^6^ CFU/ml was added to give a final volume of 200 *μ*L to each well. The wells with broth and 2.5% DMSO served as sterile control. The preincubation absorbance readings of the plate were measured at 590 nm using a microplate reader (Tecan GENios-Pro microplate reader, Grödig, Austria) before incubation, and the plate was incubated overnight without shaking at 37°C in a LAB Doctor Mini Incubator (MID SCI, USA). Cell density was determined after incubation for 18–20 hours. Cell viability was determined by finding the difference between preincubation and postincubation values. Data are presented as percentage inhibition of bacteria. The percentage inhibition was obtained using the following equation:

(2) $$\begin{array}{c} \%\text{ inhibition}=\frac{\text{Control }\left(\text{untreated cells}\right)-\text{sample value}}{\text{Control }\left(\text{untreated cells}\right)}\times 100. \end{array}$$

Cell viability after exposure to the extract was further evaluated by adding 40 *μ*l of 0.2 mg/mL of 2-(4-iodophenyl)-3-(4-nitrophenyl)-5-phenyl-2*H*-tetrazolium chloride (INT) to each of the wells of the plate, and incubating the plate at 37°C for 2 hours. Viable bacteria turn the colourless INT to red. The lowest concentration of the extract that completely inhibited bacterial growth during overnight incubation was determined to be the minimum inhibitory concentration (MIC). In the INT assay, wells with an extract concentration that prevented colour change were considered to be the MIC. The minimal bactericidal concentration (MBC) of the extract was determined by streaking a loopful of bacteria from wells with the ×½, ×1, and ×2 MIC concentrations on tryptic soy agar plates. The plates were incubated overnight at 37°C, and the MBC was determined. The least concentration of the extract lacking visible bacterial colonies was considered to be MBC.

### 2.6. Effects of *C. apiculatum* Acetone Extract on Protein Leakage

The amount of proteins that leaked from *A. baumannii* after exposure to the *Combretum apiculatum* acetone (CAA) extract was determined according to the method by Du et al. [31] with some modifications. Cells were grown overnight for 20 hours at 37°C in a Lab-Companion incubator (S1300 shaker incubator, Jeiotech, Korea). They were harvested by centrifugation and suspended in 0.9% saline solution to give OD_600_ 1.5. The cell suspension was exposed to the extract concentration of MIC (125 *µ*g/mL), × ½ MIC (62.5 *µ*g/mL), and ×2 MIC (250 *µ*g/mL). Controls used were standard drug polymyxin B sulfate, 0.1% SDS, *A. baumannii*, and DMSO. The samples were incubated at 37°C with shaking (100 rpm) for 2 hours in a Lab-Companion incubator (S1300 shaker incubator, Jeiotech, Korea). Cell suspension aliquots of 500 *μ*l were centrifuged at 4000 rpm for 4 minutes. To 50 *μ*l of the supernatant, 950 *μ*l of Bradford reagent was added to determine the protein content using the Bradford method. Bovine serum albumin (BSA) was used as a standard protein in the Bradford assay. The amount of protein leaked from the cells was quantified by measuring absorbance at 590 nm reading using the microplate reader (Tecan GENios-Pro microplate reader, Grödig, Austria).

### 2.7. The Effects of *C. apiculatum* Acetone Extract on Nucleic Acid Leakage

The potential of the CAA extract to cause nucleic acid leakage from *A. baumannii* was determined using propidium iodide as described by El-Nakeeb et al. [32] with some modifications. Propidium iodide is a red fluorescent nucleic acid stain that intercalates with the DNA of dead cells, as it penetrates only cells with disrupted membranes and is generally excluded from viable cells [33]. Cells grown overnight were centrifuged and suspended in 0.9% saline solution (OD_600_ 1.5). The suspension of *A. baumannii* cells was exposed to extract concentration of ×½ MIC (62.5 *µ*g/mL) MIC (125 *µ*g/mL) and ×2 MIC 2 (250 *µ*g/mL). Untreated cells, 0.1% SDS, 3%DMSO, and polymyxin B sulfate were used as controls. Samples were incubated at 37°C with shaking (100 rpm) for 30 minutes in a Lab-Companion incubator (S1300 shaker incubator, Jeiotech, Korea). For each 1 mL sample, the aliquots were centrifuged at 1100 rpm for 1 minute in a microcentrifuge (Eppendorf™ 5415 C). The pellet was washed with 1 mL 0.9% saline solution and suspended in 3 mL of saline. A volume of 3 *µ*L propidium iodide was added to each sample, and the solution was mixed. The samples were kept in the dark for 10 minutes after which fluorescence was measured at excitation and emission wavelengths of 544 and 612 nm, respectively, using an *f*_max_ microplate spectrofluorometer (Molecular Devices, Sunnyvale, USA).

### 2.8. The Effect of the Extracts on Biofilms

The antibiofilm activities of the extracts of CAA, CAM, CZM, and EAM were determined by staining the biofilms using crystal violet according to O'Toole [34].

#### 2.8.1. The Effect of Extracts on Biofilm Formation

*A. baumannii* cells grown overnight were centrifuged at 3500 rpm for 5 minutes (Hettich Rotofix 32 centrifuge (Tuttlingen, Germany). The remaining pellet was washed with PBS (0.5 M, pH 7.2). Cells were standardised using the 0.5 McFarland's standard to a concentration of 2 × 10^6^ CFU/ml. A volume of 1 ml of cells was added to each of the wells of a 24-well microplate. The cells were allowed to adhere to the plate by incubating them in a shaking incubator at 37°C for 2 hours.

Stock concentrations of the extracts were prepared. An equal volume of extracts (1000 *µ*g/mL) was added to the wells of the plate. The 24-well microplate was incubated for 72 hours at 37°C on a LAB Doctor Mini Incubator (MID SCI, USA). After incubation, nonadherent cells were removed by gently washing 3 times with PBS. The plates were dried by inverting them on absorbent paper for 15 minutes. The samples were fixed at 60°C for an hour in a drying oven (Mermmet Universal oven, southern Germany). The biofilms were stained with crystal violet. The plate was gently washed with sterile water and left to dry overnight. A volume of 2.5 mL ethanol was added to each well, and 200 *µ*L of the plate contents were transferred to a 96-well microplate. Optical density readings were taken at 590 nm. The percentage biofilm growth was calculated using the following formula:

(3) $$\begin{array}{c} \%\text{ Biofilm growth}=\frac{\text{ODsample}}{\text{OD control}}\times 100. \end{array}$$

#### 2.8.2. Microscopic Analyses of *A. baumannii* Static Biofilms

Microscopic analysis of the effects of CAA, CAM, CZM, and EAM on *A. baumannii* biofilm structure was carried out by staining the biofilms with a crystal violet stain and visualising these under a digital microscope. An overnight culture of *A. baumannii* was standardised to 2 × 10^6^ CFU/mL using a 0.5 McFarland standard. The extracts (1000 *μ*g/mL) and cells were dispensed in a ratio of 10 mL: 10 mL in Petri plates containing a sterile microscopic glass slide. Petri plates were incubated at 37°C for 72 hours. A plate with Muller–Hinton broth was included as a control for sterility. A plate with cells not exposed to the extract served as a positive control. After the incubation period, 2.7 ml of 1.5% SDS in PBS (v/v) was added to the plates. The plates were incubated for another 30 minutes at 37°C. Microscopic slides were removed from the Petri plates aseptically and washed with PBS. The bacterial biofilms were fixed to the slides using 2% sodium acetate and stained with 0.1% crystal violet. The slides were washed and air-dried. After drying, the biofilms were visualized under a Celestron digital microscope (Celestron, California, USA) at ×40 magnification.

#### 2.8.3. Evaluation of the Biofilm Disruption Potential of Potent Extracts on Mature *A. baumannii* Biofilms

A 24-well plate was prepared as described in the static biofilm formation assay without the addition of the test sample (extracts). The plate was incubated for 72 hours at 37°C on a LAB Doctor Mini Incubator (MID SCI, USA). After incubation, cells were washed and the test sample (1000 *µ*g/mL) was dispensed into the wells. The plate was further incubated for 24 hours, and the biofilms were quantified following the procedure for static biofilms.

#### 2.8.4. Microscopic Analysis of the Effect of Potent Extracts on Mature *A. baumannii* Biofilms

Microscopic analysis of the effects of the extracts of CAA, CAM, CZM, and EAM on mature biofilm structure was carried out as described in the microscopic analysis of the static biofilm formation assay without the addition of the extracts. Petri plates were incubated at 37°C for 72 hours. After the incubation period, the microscopic slides were washed gently using PBS to remove nonadherent cells. A volume of 10 mL of the test sample (1000 *µ*g/mL) was distributed into the Petri plates containing the microscopic slides. The Petri dishes were incubated for 24 hours. After further incubation, the slides were prepared according to the microscopic analysis of the static biofilm formation assay procedure and visualized under a Celestron digital microscope (Celestron, California, USA) at ×40 magnification.

### 2.9. Evaluation of the Toxicities of Potent Extracts

*In vitro* toxicity evaluations of CAA, CAM, CZM, and EAM extracts were investigated using the haemolysis assay and toxicity test on mouse peritoneal cells.

#### 2.9.1. Toxicity Determination Using Haemolysis Assay

**Figure 1:**
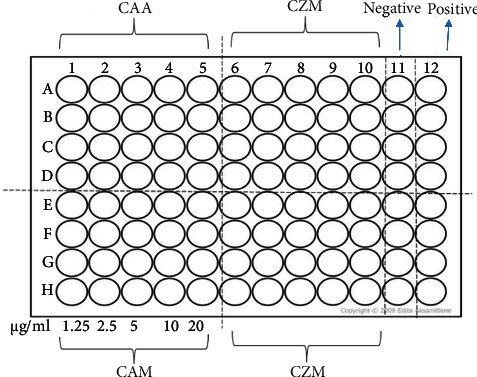
Haemolysis template plate for the effects of the extracts on sheep erythrocytes. Illustrative representation of the plate layout set to determine the haemolysis of sheep erythrocytes exposed to extracts of *C. apiculatum* acetone (CAA), *C. apiculatum* methanol (CAM), *C. zeyheri* methanol (CZM), and *E. abyssinica* methanol (EAM). All extracts ranged from 1.25, 2.5, 5, 10, and 20 mg/mL. The negative control contained centrifuged cells in Drabkin's reagent. A positive control with 100% haemolysis was obtained by mixing uncentrifuged erythrocytes with Drabkin's reagent.

The haemolysis assay was performed as described by Malagoli [35]. Blood (20 mL) was aseptically collected from an adult sheep in the animal house (the University of Zimbabwe). An equal volume of Elsevier's solution, an anticoagulant, was immediately added. Blood was centrifuged (Hettich Rotofix 32 centrifuge, Tuttlingen, Germany) at 3000 rpm for 10 minutes, and the supernatant was discarded. The remaining residue was washed three times with PBS. The washed cells were diluted four times with PBS. Cells (500 *µ*L) were incubated with an equal volume of varying concentrations of extract dissolved in PBS for 90 minutes at 37°C. After incubation, the tubes were spun in a microcentrifuge (Eppendorf™ 5415 C) at 3000 rpm for 1 min. The resulting supernatant (200 *µ*L) was added to 3 mL of Drabkin's reagent. An uncentrifuged mixture of erythrocyte suspension and PBS was a positive control. The centrifuged erythrocyte suspension and buffer (PBS) were the negative control. A volume of 200 *µ*L of supernatant aliquots was placed in Drabkin's reagent on 96-well microplates (Figure 1).

Absorbance readings of the samples taken using a Tecan GENios-Pro microplate reader (Grödig, Austria) at 590 nm were used to estimate the amount of haemoglobin released. The percentage of haemolysis for each sample was calculated using the following formula:

(4) % haemolysis= sample's absorbance−negative control absorbancepositive control absorbace−negative control absorbance×100.

#### 2.9.2. Toxicity Tests in Mouse Peritoneal Cells

Male mice (BALB c) of 20–25 g weight were collected from the animal house (the University of Zimbabwe). The starch solution (20%) was injected intraperitoneally into 2 mice to increase the production of peritoneal cells within the mice. The mice were left for 48 hours in plastic cages with an unlimited pelleted basal diet and water. The peritoneal cells were isolated according to a method described by Ray and Dittle, [36]. The mice were sacrificed using the cervical dislocation method. Each mouse was then sprayed with 70% ethanol and mounted on a Styrofoam board on its back using pins. The outer skin of the peritoneum was gently cut and pulled using scissors and forceps to expose the inner peritoneal cavity. A volume of 5 mL cold FBS (3%) and a solution of PBS were injected into the peritoneal cavity without damaging any organs. The peritoneum was gently massaged with fingers to allow the attached cells to enter the buffer solution. A pasture pipette was used to collect as much fluid as possible. The fluid collected was kept on ice. Cells harvested were centrifuged in a Hettich Rotofix 32 centrifuge for 10 min at 1500 rpm [37]. The cell pellet was collected and suspended in RPMI supplemented with 10% FBS and 1% PNS (penicillin, neomycin, and streptomycin). Cells were incubated overnight at 37°C in a CO_2_, series II water-jacket incubator (Thermo Forma, Ohio, USA).

Mouse peritoneal cell viability was determined using a trypan blue exclusion assay. Cells were stained with 0.4% trypan blue and manually counted using a haemocytometer beneath a Celestron digital light microscope (Celestron, California, USA) using a ×10 objective lens. The percentage of cell viability was 98%.

**Figure 2:**
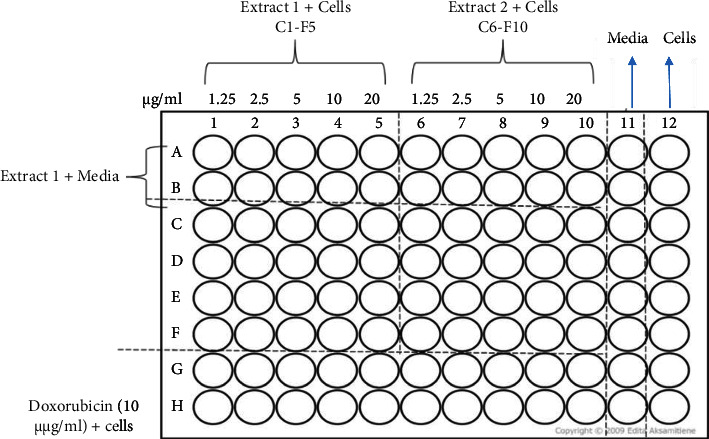
A 96-well template for toxicity testing using mouse peritoneal cells. Controls were cells only (negative control) and cells exposed to doxorubicin served as the positive control. Doxorubicin (10 *µ*g/mL) and plant extracts (1.25–20 mg/mL) were tested against peritoneal cells.

The MTT assay described by Kundishora et al. [38] was used to determine toxicity. The extracts of CAA, CAM, CZM, and EAM were prepared to give concentrations of 1.25, 2.5, 5, 10, and 20 mg/ml. Each well contained 100 *μ*L of test extract and 100 *μ*L of 0.5 × 10^5^ cells/mL in supplemented RPMI. Cells exposed to the standard anticancer drug doxorubicin (10 *µ*g/mL) were used as a positive control. Cells in RPMI were used as negative control. Cells were incubated in 96-well plates (Figure 2) in the presence of extracts for 24 h at 37°C in a CO_2_, water-jacketed incubator series II (Thermo Forma, Ohio, USA).

After incubation, 40 *µ*L of MTT (3-(4, 5- dimethylthiazol-2-yl)-2, 5-diphenyltetrazolium bromide) was added to each well and the plate was incubated for 2 hours. A volume of 50 *µ*L of DMSO was added, and the optical density at 590 nm was measured using a microplate reader (Tecan GENios-Pro microplate reader, Grödig, Austria).

### 2.10. Statistical Analysis

Graph Pad prism for windows (Graph Pad Software Inc., San Diego, California, USA) version 8.0.1 was used to analyse data from the results obtained in this study. The one-way analysis of variance test (ANOVA) with Dunnett's multiple comparison test was used to determine the level of significance, where all treated samples were compared to the control. Statistical significance was considered if *P* values < 0.05.

## 3. Results

### 3.1. Antibacterial Activities of Extracts

**Figure 3:**
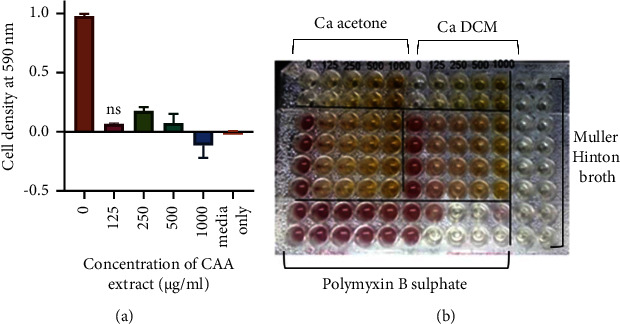
The effects of the most potent extracts of *C. apiculatum* on the growth of *A. baumannii*: (a) are the data analysed and (b) is a picture of the effects of the acetone and DCM extracts. Extract concentrations ranged from 0 to 1000 *μ*g/mL. Bacteria used were 2 × 10^6^ CFU/mL. Values are expressed as mean cell density at a wavelength of 590 nm wavelength ± the standard deviation (*n* = 4). The two extracts of *C. apiculatum* had a significant effect on the growth of *A. baumannii.*

Extracts from 8 medicinal plants from Zimbabwe prepared from solvents of varying polarities were screened for antibacterial activity against *A. baumannii*. Polymyxin B sulfate was used as a positive control. The standard drug had a MIC of 2 *µ*g/mL. A total of 7 out of 8 medicinal plants evaluated for antibacterial activity were found to have an antibacterial effect against *A. baumannii*. The leaf extracts that showed the highest activity against *A. baumannii* for each plant are shown in Table 2. The CAA extract was the most potent with an MIC of 125 *µ*g/mL (Figure 3). Other potent extracts included the CAM extract with a MIC of 250 *µ*g/mL, EAM extract (MIC-500 *µ*g/mL), *S. guineense* ethyl (SGE) acetate extract (MIC- 500 *µ*g/mL), and CZM extract (MIC-1000 *µ*g/mL).

### 3.2. The Effects of *C. apiculatum* Acetone Extract on the Membrane Integrity of *A. baumannii*

**Figure 4:**
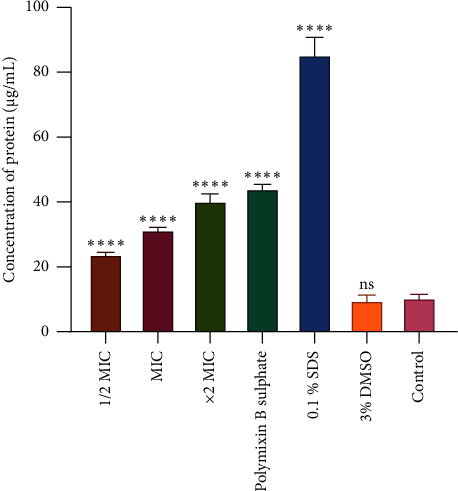
The effect of *Combretum apiculatum* acetone (CAA) extract on protein leakage from *A. baumannii* cells. Cells were exposed to CAA at ×½ MIC, ×1 MIC, and ×2 MIC concentrations and controls. The protein leaked from cells is shown in *µ*g/mL. Polymyxin B sulfate and 0.1% SDS were used as positive controls. Untreated cells were used as the negative control. The values are for the mean ± standard deviation (error bar) for *n* = 4. The asterisks indicate a significant difference from the control (untreated cells) with ^∗∗∗∗^*P* < 0.0001. The extract caused protein leakage of 24, 32, and 41 *µ*g/mL at ½ MIC, MIC, and ×2 MIC concentrations, respectively.

The ability and extent of the CAA extract to cause membrane damage to *A. baumannii* as a mode of action were assessed using the protein leakage and nucleic acid assay. In the protein leakage assay, protein content was estimated using the bovine serum albumin (BSA) standard curve after *A. baumannii* were exposed to varying concentrations of the extract, as shown in Figure 4. The extract managed to cause leakage of protein from the concentrations of ½ MIC, MIC, and ×2 MIC. The protein concentration leaked was 24, 32, and 41 µg/mL for ½ MIC, MIC, and ×2 MIC concentrations, respectively.

**Figure 5:**
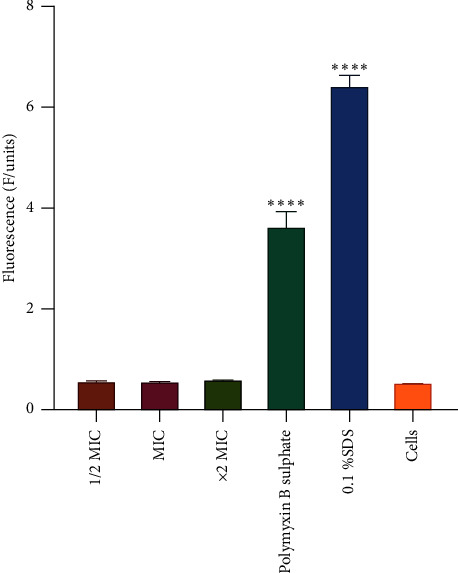
The effect of *Combretum apiculatum* acetone (CAA) extract on nucleic acid leakage from *A. baumannii* cells. Polymyxin B sulfate and 0.1% SDS were used as positive controls. Untreated cells were used as the negative control. The values are for mean ± standard deviation (error bar) for *n* = 4. The asterisks indicate a significant difference from the control (untreated cells) with ^∗∗∗∗^*P* < 0.0001. The acetone extract did not cause significant leakage of nucleic acids from *A. baumannii.*

In the nucleic acid assay, the fluorescence of propidium iodide of the different samples exposed to the CAA extract is shown in Figure 5. Acetone extract did not cause significant leakage of nucleic acids from *A. baumannii* compared to unexposed cells, as the fluorescence of propidium iodide was comparable to that of the control. An increased fluorescence of propidium iodide was observed in cells treated with polymyxin B and 0.1% sodium dodecyl sulfate.

### 3.3. Determination of the Effect of Potent Extracts on the Formation of *A. baumannii* Biofilms

**Figure 6:**
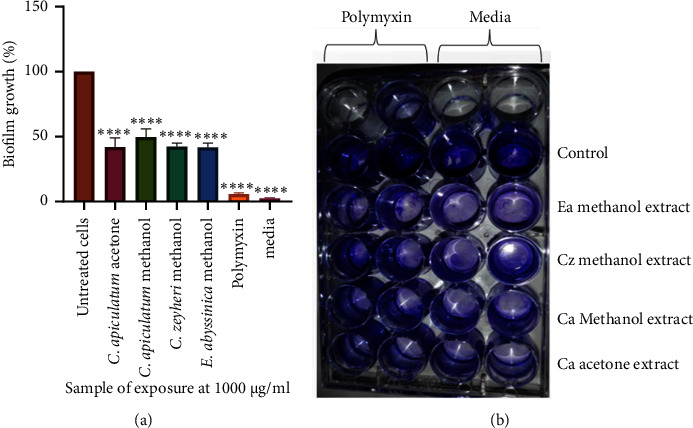
Qualitative analysis of the effects of extracts and polymyxin B sulfate on biofilm formation in *A. baumannii*. (a) The negative control contained only broth. The error bars indicated the standard deviation from the mean (*n* = 4). The asterisks ^∗^indicate statistically significant differences compared to the positive control (unexposed cells), which represented cells without any extract, where ^∗∗∗∗^ denotes *P* < 0.0001. At 1000 *μ*g/mL, extracts from CAA, CAM, CZM, and EAM resulted in only 42%, 50%, 42% and 41% biofilm formation by *A. baumannii,* respectively. Slide (b) shows a picture of the 24-well plate used in the assay.

Antibiofilm activities of the extracts of CAA, CAM, CZM, and EAM were determined by quantifying the biofilms using crystal violet. All extracts inhibited the formation of biofilms of *A. baumannii.* There was a significant difference in the biofilm formed when cells were exposed to the extracts compared to the biofilm formed in the unexposed cells. At 1000 *μ*g/mL, the extracts of CAA, CAM, CZM, and EAM resulted in only 42%, 50%, 42%, and 41% biofilm formation by *A. baumannii,* respectively **(**Figure 6**)**.

### 3.4. Microscopic Analyses of Static *A. baumannii* Biofilms

**Figure 7:**
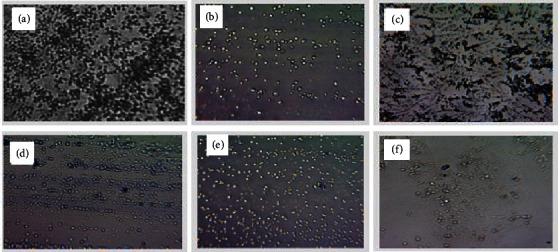
Microscopic visualisation of the effects of extracts and polymyxin B sulfate on the formation of biofilms in *A. baumannii*. Images of the effects of untreated *A. baumannii* cells (a), cells treated with polymyxin B sulfate (b), *C. apiculatum* acetone (CAA) (c), *C. apiculatum* methanol (CAM) (d), *C. zeyheri* methanol (CZM) (e), and *E. abyssinica* methanol (EAM) (f) extracts on *A. baumannii* biofilm formation under a Celestron digital microscope at 40x magnification. Cells treated with standard drugs or extracts showed dispersed planktonic cells. Untreated cells (a) showing an undisturbed biofilm structure were used as the control.

Microscopic analysis of the effects of CAA, CAM, CZM and EAM extracts on the formation of *A. baumannii* biofilms was carried out by staining the biofilms using a crystal violet stain and viewing the microscopic sides at ×40 magnification using a Celestron™ digital microscope (Celestron, LLC, California, USA). *A. baumannii* cells exposed to standard drug or extracts (CAA, CZM, and EAM) showed dispersed planktonic cells. Cells exposed to the CAM extract showed a partially disrupted biofilm structure. Unexposed cells of *A. baumannii* showed an undisturbed biofilm structure (Figure 7).

### 3.5. Evaluation of the Biofilm Disruption Potential of Extracts in Mature Biofilms

**Figure 8:**
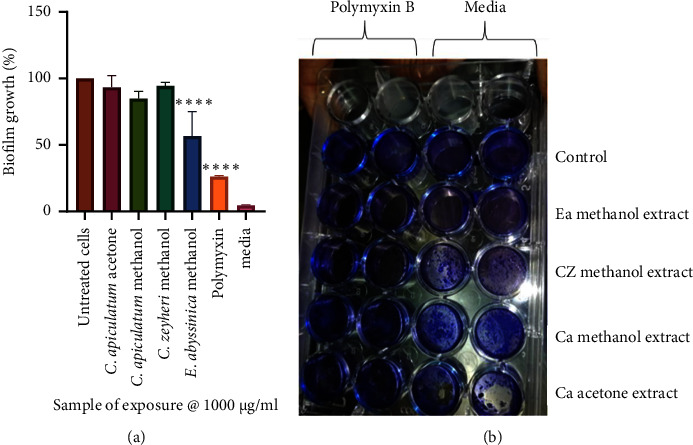
Qualitative analysis of the effects of extracts and polymyxin B sulfate on the formation of mature biofilms in *A. baumannii.* (a) The negative control contained only broth. The error bars indicated the standard deviation from the mean (*n* = 4). The asterisks () indicate statistically significant differences compared to the positive control (unexposed *A. baumannii*), which represented cells without any extract, where ^∗∗∗∗^ denotes *P* < 0.0001. Exposure of cells to CAA, CAM, and CZM extracts did not show antibiofilm activity on mature biofilms. Exposure to the EAM extract resulted in the reduction of mature biofilms. Slide (b) shows a picture of the 24-well plate used in the assay.

The potential of the extracts to disrupt mature biofilms was evaluated using the crystal violet assay. The CAA, CAM, and CZM extracts did not show antibiofilm activity on mature biofilms. Exposure of mature biofilms to the EAM extract resulted in reduction of biofilms. Only 57% biofilm growth was observed **(**Figure 8**)**.

### 3.6. Microscopic Analysis of Mature Biofilms

**Figure 9:**
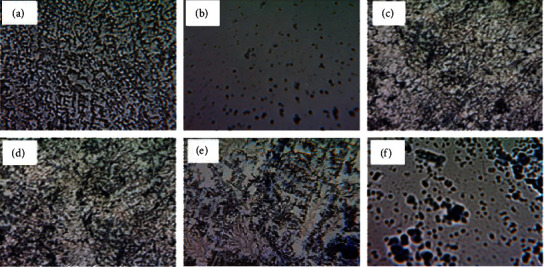
Microscopic visualisation of the effects of extracts and polymyxin B sulfate on the formation of mature biofilms in A. baumannii. Images of the effects of untreated A. baumannii cells (a), cells treated with polymyxin B sulfate (b), C. apiculatum acetone (CAA) extract (c), C. apiculatum methanol (CAM) extract (d), C. zeyheri methanol (CZM) extract (e) and E. abyssinica methanol (EAM) extract (f) on mature biofilms of A. baumannii under celestron digital microscope at ×40 magnification. The cells treated with the extracts showed a compact biofilm structure that was comparable to the untreated cells (a), except for the cells treated with the E. abyssinica methanol extract (f). A partial biofilm disruption was observed in image (f). Biofilm structures were not observed in cells treated with polymyxin B, image (b).

The potential disruption of mature biofilms by the CAA and CAM, CZM, and EAM extracts in mature biofilms of *A. baumannii* was determined. The effects were observed on the slides under a Celestron^TM^ digital microscope (California, USA) at a ×40 magnification. When mature biofilms were exposed to the EAM extract, a partial biofilm disruption was observed. The mature biofilms of *A. baumannii* treated with CAA, CAM, and EAM extracts showed a compact biofilm structure. Biofilm structures were not observed in biofilms exposed to polymyxin B **(**Figure 9**)**.

### 3.7. *In Vitro* Toxicity: Haemolysis Assay

**Figure 10:**
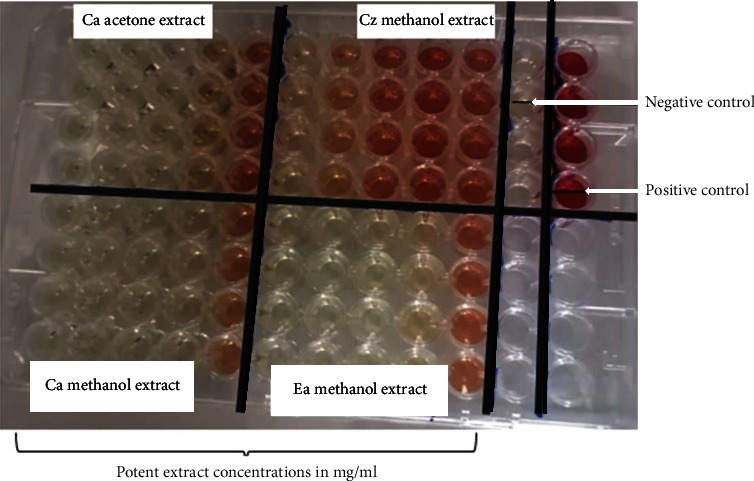
The effects of exposing sheep erythrocytes to the extracts. A 96-well microplate was used to determine the haemolysis activity of sheep erythrocytes exposed to extracts of *C. apiculatum* acetone (CAA), *C. apiculatum* methanol (CAM), *C. zeyheri* methanol (CZM), and *E. abyssinica* methanol (EAM). The negative control contained centrifuged cells in Drabkin's reagent. A positive control with 100% haemolysis was obtained by mixing uncentrifuged erythrocytes with Drabkin's reagent. The intensity of the colour increased with an increase in the extract concentration of 1.25, 2.5, 5, 10, and 20 mg/ml.

**Figure 11:**
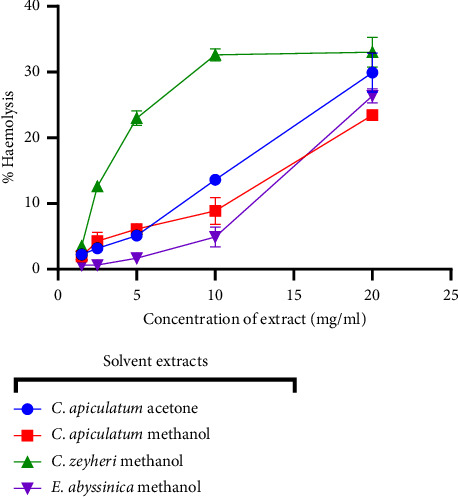
Quantitative determination of the effects of exposing sheep erythrocytes to the extracts. The haemolysis effect of CAA, CAM, CZM, and EAM on sheep erythrocytes. The erythrocytes were exposed to varying concentrations of extracts. Haemolytic activity of 30%, 23%, 33%, and 26.4% was observed for the highest concentration (20 mg/mL) of the CAA, CAM, CZM, and EAM extracts, respectively.

The effect of the extracts of CAA, CAM, CZM, and EAM on sheep erythrocyte membrane integrity was investigated using the Drabkin cynamethemoglobin method. The activity of the four extracts is expressed as a percentage haemolysis. The effect of the extracts on sheep erythrocytes is shown in Figure 10. There was an increase in haemolysis activity as the extract concentration increased. The intensity of the colour increased with increasing release of haemoglobin. Plant extracts are deemed toxic to red blood cells if the degree of haemolysis is greater than 30% (ISO 100993−5, 2009). CAA, CAM, and EAM extracts were considered nontoxic since they showed a percentage haemolysis of 30%, 23%, and 26.4% at 20 mg/mL, respectively. The CZM extract showed low toxicity with a percentage haemolysis of 33% at 20 mg/mL, as seen in Figure 11.

### 3.8. Effect of CAA Extract on Mouse Peritoneal Cells

**Figure 12:**
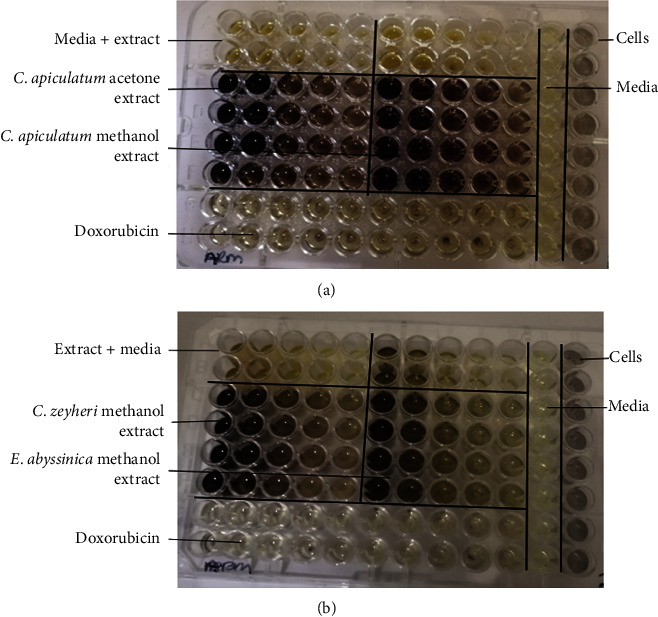
The effect of the extracts on mouse peritoneal cells. An image of a 96-well plate used for the MTT assay for (a) *C. apiculatum* extracts of acetone (CAA) and methanol (CAM) and (b) extracts of *C. zeyheri* methanol (CZM) and *E. abyssinica* methanol (EAM). Controls were cells only (negative control) and doxorubicin (positive control). The intensity of the colour increased with increasing extract concentration. Only wells from the media + extract were used to subtract the effect of coloured extracts.

**Figure 13:**
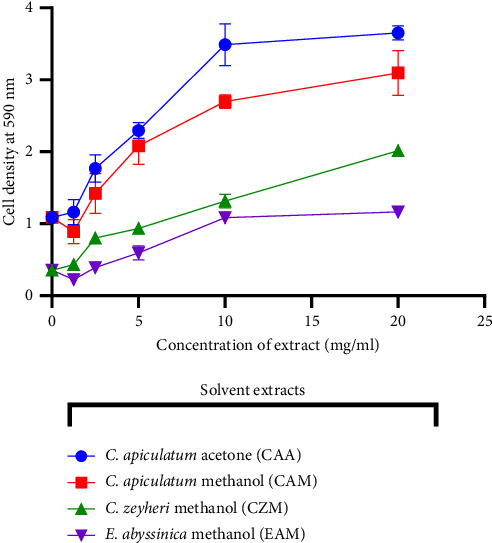
Quantitative determination of the effects of exposing mouse peritoneal cells to the extracts. The cytotoxic effect of the *C. apiculatum* acetone (CAA), *C. apiculatum* methanol (CAM), *C. zeyheri* methanol (CZM), and *E. abyssinica* methanol (EAM) extracts in mouse peritoneal cells. Cell density increased with increasing extract concentration. The values are for mean ± standard deviation (error bar) for *n* = 4. The asterisks indicate a significant difference from the control ^∗∗∗∗^*P* < 0.0001. There was ×3.4, ×3, ×6, and ×3.6 fold increase in cells as a result of exposure to CAA, CAM, CZM, and EAM extracts, respectively.

The cytotoxicity effect of four extracts that showed antibacterial activity against *A. baumannii* was tested on immune cells elicited from the peritoneal cavities of mice. The effect was determined using the MTT assay. The yellow tetrazolium MTT salt was reduced by dehydrogenase enzymes in metabolically active cells, giving a purple colour [39]. The intensity of the purple colour was used to calorimetrically measure viable cells. As can be seen in Figure 12, the four extracts were not toxic to immune cells. Exposure to the extract resulted in an increase in cell density with an increase in extract concentration. All extracts significantly increased peritoneal cell viability with the CZM extract giving the most increase of 6 fold **(**Figure 13**)**. The media+extract-only wells were used to subtract the effect of coloured extracts.

## 4. Discussion

The increase in microbial resistance to conventional antibiotics has raised serious concerns in the treatment of infectious diseases [40]. *A. baumannii* is one of the serious multidrug-resistant pathogens. The need to find new potential antibacterial compounds against MDR *A. baumannii* is urgent [41]. Plants are a valuable source of new bioactive compounds with antimicrobial activities for pharmaceutical development [42]. They are effective in the treatment of infectious diseases, while at the same time alleviating many of the side effects that are often associated with synthetic antimicrobial agents [43].

The study aimed to evaluate the antibacterial activities of extracts of eight medicinal plants in Zimbabwe against a first-priority pathogen of the WHO *A. baumannii*. A total of seven out of eight medicinal plants evaluated for antibacterial activity were found to have an antibacterial effect against *A. baumannii*. The serial exhaustive extraction gave the best result compared to extractions with DCM-methanol or water-ethanol solvents. The CAA extract was the most potent inhibitor of the growth of *A. baumannii.* Other extracts that were more potent included CAM, EAM, SGE, and CZM extracts. The crude extracts that were potent inhibitors of growth were extracted using polar solvents. Polar solvents are known to extract alkaloids, flavonoids, terpenoids, saponins, and tannins [44], which may be attributed to the antibacterial activity observed in this study. The choice of solvent and the sequence of extraction in natural products preparation depend on the specific compounds you aim to extract and their solubility properties. Generally, extraction can be done in a sequence from nonpolar to polar or polar to nonpolar, depending on the desired outcomes [25]. Based on our results, we think it is best to have the 8 extracts in a sequential order ranging from nonpolar to polar as it will be easier to remove the organic solvents because of their low boiling points under reduced pressure. There is a need for improvement of the solvent ratio so as to optimize the ratio of solvent to plant materials to ensure complete extraction of target compounds and to make sure that each extraction step is performed thoroughly before moving to the next solvent [45]. There could be a need for ultrasonication to enhance solvent penetration as this may improve extraction efficiency.

In a study by Mangoyi et al. [20], Combretum species including *C. apiculatum* and *C. zeyheri* were found to possess inhibitory activity against *C. albicans* and *C. krusei*. When phytochemical identification was carried out, flavonoids were found to have the most antifungal activity. In other studies, the *C. apiculatum* acetone extract exhibited anti-inflammatory and anthelmintic activity [46]. In another study, volatile oils from cinnamon, clove, and tree basil were found to possess antibacterial activity against MDR *A. baumannii* with MBC of 0.5, 1, and 2 mg/mL [47]. The study used an increased concentration of extracts compared to our study where our highest concentration was 1000 *µ*g/mL. Studies by Miyasaki et al. [48] demonstrated that a pure compound norwoginin, a flavone extracted from *Scutellaria baicalensis,* had significant antibacterial activities against *A. baumannii* with a MIC of 126 *µ*g/mL. According to the literature, the common type of compounds in *C. molle*, *C. apiculatum*, *C. zeyheri*, and *S. guineense* are flavonoids particularly quercetin and kaempferol. In addition, tannic acid and ellagitanins are also found in all the 4 species [49]. In our own studies, we were able to isolate *β*-sitosterol from both *P. curatellifolia* and *C. platypetalum* [50, 51]. The chemical components of the extracts were not compared in this study as it was initial screening against this number 1 priority pathogen to determine which plant species would produce antibacterial bioactive components.

Secondary metabolites from plants can affect microbial cells by altering and permeabilizing the membrane structure [52]. The ability and extent of CAA to cause membrane damage of *A. baumannii* as a mechanism of action using the protein and nucleic acid leakage assay. Exposure of cells to × ½ MIC, ×1 MIC, and ×2 concentrations of the CAA extract resulted in an increased leakage of proteins indicating membrane disruption. In the nucleic acid assay, the acetone extract did not cause significant leakage of nucleic acids from *A. baumannii* compared to untreated cells. In a similar study, Hao et al. [53] demonstrated that *Litsea cubeba* essential oil can disrupt the integrity of the *A. baumannii* membrane and contribute to the leakage of nucleic acids and proteins. Leakage of cell components can lead to inhibition of bacterial growth [54]. Other studies have reported the elimination of reactive oxygen species and protein leakage as a phytochemical-induced action mechanism against *A. baumannii* [54, 55]. The results of these studies can explain why we only observed protein leakage and did not observe nucleic acid leakage in this study.

*A. baumannii,* a leading nosocomial pathogen, can persist in hospital environments and medical devices due to its ability to form biofilms [56]. Biofilms can be defined as a complex community of microbes that can be found attached to a surface or form aggregates [57]. The formation of these biofilms is one of the leading causes of multidrug resistance in bacteria and ultimately increases the cost of treating patients [58]. The integrity of cells in the biofilm matrix limits the permeability of antibiotics [55]. In the study, the inhibitory activity of the biofilm formation and the disruption potential of mature biofilms of CAA, CAM, CZM, and EAM extracts against *A. baumannii* were investigated. All four extracts inhibited the formation of biofilms in *A. baumannii.* This work is in contract with our previous study [59] in which we showed that the extracts and a pure compound of tormentic acid, isolated from *Callistemon viminalis,* had no effect on *A. baumannii*. There was a significant difference in the biofilm formed when cells were exposed to the extracts compared to the biofilm formed in the unexposed cells. Biofilms are formed in four stages which consist of an attachment of bacteria to the surface, microcolony formation, maturation of biofilms, and dispersal of bacteria to find new niches [60, 61]. When observed under a microscope, *A. baumannii* cells exposed to the extracts could be seen as planktonic cells compared to the untreated cells that showed a compact biofilm structure. Therefore, the extracts inhibited the formation and maturation of microcolony in the biofilms. Other studies have indicated that the most effective treatment for biofilm-based infections is to inhibit the initial binding phase [58]. Therefore, inhibition of the initial biofilm formation by plant extracts may provide a strategy for the prevention and treatment of biofilm-based infections caused by *A. baumannii*.

When the disruption potential of the extracts was investigated in mature biofilms, only the EAM extract showed activity in mature biofilms. Polar solvents such as methanol have been shown to extract the highest amount of bioactive compounds [62]. The antibiofilm properties shown by the EAM extract may be attributed to the alkaloids, tannins, flavonoids, and saponins found in the plant [23]. Similarly, in a study by Sanchez et al. [63], methanolic extracts of *Opuntia ficus-indica* caused a reduction in biofilm formation against *A. baumannii* biofilms. Phytochemical analysis revealed the presence of flavonoids, coumarins, and tannins. In other studies by Karunanidhi et al. [64], *Alium stipitatum* hexane and DCM extracts were found to have antibiofilm activity against *A. baumannii* and methicillin-resistant *S. aureus.* The antibiofilm activity of natural products is mainly based on suppression of cell adhesion and attachment, inhibition of polymer matrix formation, and decreasing virulence factor production, thus blocking the quorum sensing network and biofilm development [55, 65]. The extracts might have exerted their antibiofilm activity using one of these mechanisms of action.

Although plant extracts can be active against microorganisms [66], they can be toxic at high doses [67]. It is necessary to conduct a toxicity study to ensure the safety of the plants in animal models [68]. The haemolytic activity of any compound is an indicator of general cytotoxicity to normal healthy cells. Haemolysis is characterised by the release of haemoglobin due to lysis of the lipid bilayer membrane [69]. The effect of CAA, CAM, CZM, and EAM extracts on sheep erythrocyte membrane integrity was investigated using the Drabkin cynamethemoglobin method. Haemoglobin from haemolysed cells is oxidized to methemoglobin, then cynamethemoglobin gives a red colour that absorbs at 590 nm [70]. The intensity of the colour is proportional to the haemoglobin released. In this study, the intensity of the colour increased with increasing extract concentration. According to the International Organization for Standardization, plant extracts are considered toxic to red blood cells if the percent haemolysis is greater than 30% as indicated by ISO100993−5 [71]. Three of the extracts tested were deemed nontoxic since the percentage of haemolysis was 30% and below. The CZM extract had a haemolytic activity of 33% and was considered slightly toxic to sheep erythrocytes. The three extracts CAA, CAM, and EAM were considered suitable for the preparation of herbal cream, since they were nontoxic at higher concentrations of 20 mg/mL.

Macrophages are immune cells that participate in both innate and adaptive immunity. Peritoneal cavity macrophages are commonly used in *in vitro* assays, as they are more stable in their functionality and phenotype [72]. The presence of a large number of naïve macrophages makes the peritoneal cavity a preferred site for the collection of macrophages [36, 73]. The potential of plant extracts to inhibit murine macrophage growth was used as an indication of toxicity. The cytotoxicity effect of the extracts of CAA, CAM, CZM, and EAM was evaluated using the MTT assay. The four extracts were nontoxic to mouse peritoneal cells but instead promoted the growth and survival of these cells with increasing extract concentration compared to the untreated cells. Several studies have also shown that plant extracts are nontoxic but have an immune-stimulatory effect on murine macrophages. Adegbaju et al. [72] showed that plant extracts from *Celosia argentea* were not toxic to RAW264 macrophages and the *Celosia agentea* acetone extract showed increased cell proliferation. Mapfunde et al. [74] also had similar findings. In that study, they found that *C. zeyheri* alkaloid, saponin, and ethanol extracts were nontoxic to mouse peritoneal cells and Jurkat T-cells. Plants can be immunomodulators that can directly inhibit or stimulate the immune system [75]. Modulation of the immune system involves influencing cytokine production, lymphocyte proliferation, and macrophage stimulation [76]. The increase in cell proliferation shown by the extracts suggests that the extracts can stimulate an immune response which is essential in the fight against bacterial infections.

## 5. Conclusions

The extracts of CAA, CAM, CZM, and EAM showed the most significant antibacterial activity against *A. baumannii*. The CAA extract has shown to damage bacterial cell membranes, resulting in protein leakage. All four extracts inhibited the formation of biofilms of *A. baumannii*. The EAM extract disrupted the mature biofilm structure of *A. baumannii*. The presence of antibacterial and antibiofilm formation activities shown by the extracts indicates that the plants have potential as sources of effective antibacterial and antibiofilm formation agents against bacterial infections caused by *A. baumannii*. Toxicity studies showed that the 4 extracts are nontoxic, so further studies can be carried out in animal models without toxicity to normal cells. The study was limited to extracts and *in vitro* studies, so further studies can be carried out to determine the active compounds responsible for the antibacterial and antibiofilm activities. *In vivo* studies of antibacterial activity in rodent models are also necessary to fully investigate the antibacterial mechanism of action of the four extracts.

## Figures and Tables

**Table 1:** Zimbabwean medicinal plants evaluated for their antibacterial activity against *A. baumannii* in this study.

Family	Plant names		Voucher used	Part used	Antibacterial activity	Ethnomedicinal information
	Scientific	Local				
Combretaceae	*Combretum molle* Engl. and Diels	*Mudziyaishe*	C9E7	Leaves	++	Treatment of parasitic infections and anthelmintic activities [19]
Combretaceae	*Combretum zeyheri* Sond	*Muruka, mupembere-kono,* and *muchenja*	N6E7	Leaves	++	Fever, wound dressing antimicrobial, and stomachache [20]
Myrtaceae	*Callistemon citrinus*	—	UZ2E7	Leaves	++	—
Chrysobalanaceae	*Parinari curatellifolia* Planch. ex Benth.	*Muhacha*	C6E7	Leaves	++	Skin rashes, *tuberculosis*, chronic diarrhea, herpes zoster, and herpes simplex [21]
Myrtaceae	*Syzygium guineense*	*Mukute*	C12E7	Leaves	++	Herpes zoster, herpes simplex, and skin rashes [21]
Combretaceae	*Combretum apiculatum* Sond.	*Bonda, Chikukute,* and *Mugodo*	C1E7	Leaves	+++	Mumps, toothache, heart diseases, backache, fever, headaches, and abdominal pains [22]
Fabaceae	*Erythrina abyssinica* Lam. ex D	*Mutiti Mulunguti* (Bemba, Tongan)	UZ11E7 UZ11E4	Leaves bark	+++	Bacterial and fungal infections, malaria, leprosy, inflammatory diseases, diabetes mellitus, obesity, anaemia, snake bites, STDs, and schistosomiasis [23]
Combretaceae	*Combretum platypetalum* Welw. ex M.A. Lawson	*Bepu*	N9E7	Leaves	—	Fever, headaches, abdominal pains, sore throats, colds, coughs, pneumonia, and conjunctivitis [22]

-No inhibitory activity; + slight inhibitory activity (10–45% inhibition); ++ medium inhibitory activity (46–75% inhibition); +++ high inhibitory activity (76–100% inhibition). The first letters on the voucher samples denote the place of location. C: Centenary, UZ: the University of Zimbabwe, and N: Norton. E7 represents the leaves and E4 represents bark.

**Table 2:** Antibacterial activities against *A. baumannii* of leaf extracts from Zimbabwean medicinal plants.

Plants	Solvent of extraction (% yield)	MIC (*µ*g/mL)	MBC (*µ*g/mL)
*Combretum apiculatum* ^∗^	Hexane (2)	500 MBC	>1000
	DCM (5.11)	500 MBC	>1000
	Acetone (4.49)	125 MBC	>1000
	Methanol (11.35)	250 MBC	>1000
	DCM-methanol	—	—
	Water-ethanol	—	—
	Ethyl acetate	—	—
	Water	—	—

*Combretum molle*	DCM (3.18)	1000	—
	Acetone (2.30)	1000	—
	Ethyl acetate (1.13)	1000	—
	DCM-methanol	—	—
	Water-ethanol	—	—
	Ethyl acetate	—	—
	Methanol	—	—
	Water	—	—

*Combretum zeyheri*	Acetone (1.20)	1000	—
	Methanol (3.30)	1000	—
	Ethanol (2.80)	1000	—
	DCM	—	—
	Ethyl acetate	—	—
	DCM-methanol	—	—
	Water-ethanol	—	—
	Water	—	—

*Callistemon citrinus*	Water (2.72)	1000	—
	Acetone	—	—
	Methanol	—	—
	DCM	—	—
	Ethanol	—	—
	Ethyl acetate	—	—
	DCM-methanol	—	—
	Water-ethanol	—	—

*Erythrina abyssinica* leaf	Methanol (2.3)	500	>1000
	Acetone	—	—
	Water	—	—
	DCM	—	—
	Ethanol	—	—
	Ethyl acetate	—	—
	DCM-methanol	—	—
	Water-ethanol	—	—

*Syzygium guineense*	Ethyl acetate (3.5)	500	>1000
	DCM (1.0)	1000	—
	Acetone	—	—
	Water	—	—
	Methanol	—	—
	Ethanol	—	—
	DCM-methanol	—	—
	Water-ethanol	—	—

*Parinari curatellifolia*	Methanol (3.31)	1000	—
	Ethanol	—	—
	DCM-methanol	—	—
	Water-ethanol	—	—
	DCM	—	—
	Acetone	—	—
	Water	—	—
	Ethyl acetate	—	—

*Combretum platypetalum*	Methanol	—	—
	Ethanol	—	—
	DCM-methanol	—	—
	Water-ethanol	—	—
	DCM	—	—
	Acetone	—	—
	Water	—	—
	Ethyl acetate	—	—

> Signifies a value greater than the stated value. ^∗^ In all cases of the 4 extracts from *C. apiculatum*, the minimum bactericidal concentration (MBC) was above 1000 *µ*g/mL for all the solvent extracts, whilst acetone, methanol, DCM, and hexane extracts' MICs were 125, 250, 500, and 500 *µ*g/mL each, respectively. This shows that the extracts can inhibit the growth but are not able to kill the bacteria up to 1000 *µ*g/mL.

## Data Availability

The data used to support the findings of this study are included within the article.

## Abbreviations

CAA: *Combretum apiculatum* acetone
CAM: *Combretum. apiculatum* methanol
CZM: *Combretum zeyheri* methanol
EAM: *Erythrina abyssinica* methanol
ESKAPE: *Enterococcus faecium, Staphylococcus aureus, Klebsiella pneumoniae, Acinetobacter baumannii, Pseudomonas aeruginosa*, and *Enterobacter* species
WHO: World Health Organization
DCM: Dichloromethane
DMSO: Dimethyl sulfoxide
INT: (2–(4-iodophenyl)-3-(4-nitrophenyl)-5-phenyl-2H-tetrazolium chloride)
TSB: Tryptic soy broth
TSA: Tryptic soy agar
MHB: Muller–Hinton broth
CECT: A registered trademark of the Universitat de Valencia
EUCAST: European Committee on Antimicrobial Susceptibility Testing
MBC: Minimum bactericidal concentration
MIC: Minimum inhibitory concentration.

## References

[1] Ekwanzala M. D., Dewar J. B., Kamika I., Momba M. N. B. (2018). Systematic review in South Africa reveals antibiotic resistance genes shared between clinical and environmental settings. *Infection and Drug Resistance*.

[2] Mulani M. S., Kamble E. E., Kumkar S. N., Tawre M. S., Pardesi K. R. (2019). Emerging strategies to combat ESKAPE pathogens in the era of antimicrobial resistance: a review. *Frontiers in Microbiology*.

[3] Schultz F., Anywar G., Tang H. (2020). Targeting ESKAPE pathogens with anti-infective medicinal plants from the Greater Mpigi region in Uganda. *Scientific Reports*.

[4] Mhondoro M., Ndlovu N., Bangure D. (2019). Trends in antimicrobial resistance of bacterial pathogens in harare, zimbabwe, 2012-2017: a secondary dataset analysis. *BMC Infectious Diseases*.

[5] Shrivastava S. R. L., Shrivastava P. S., Ramasamy J. (2018). World Health Organization releases global priority list of antibiotic-resistant bacteria to guide research, discovery, and development of new antibiotics. *Journal of Medical Society*.

[6] Peleg A. Y., Seifert H., Paterson D. L. (2008). *Acinetobacter baumannii*: emergence of a successful pathogen. *Clinical Microbiology Reviews*.

[7] Eze E., Chenia H. Y., El Zowalaty M. E. (2018). *Acinetobacter baumannii* biofilms: effects of physicochemical factors, virulence, antibiotic resistance determinants, gene regulation, and future antimicrobial treatments. *Infection and Drug Resistance*.

[8] Elhosseiny N. M., Attia A. S. (2018). Acinetobacter: an emerging pathogen with a versatile secretome. *Emerging Microbes and Infections*.

[9] Han van der Kolk J., Hermanus J. (2015). *Acinetobacter baumannii* as an underestimated pathogen in veterinary medicine. *Veterinary Quarterly*.

[10] Lee C. R., Lee J. H., Park M. (2017). Biology of *Acinetobacter baumannii*: pathogenesis, antibiotic resistance mechanisms, and prospective treatment options. *Frontiers in Cellular and Infection Microbiology*.

[11] Vrancianu C. O., Gheorghe I., Czobor I. B., Chifiriuc M. C. (2020). Antibiotic resistance profiles, molecular mechanisms and innovative treatment strategies of *Acinetobacter baumannii*. *Microorganisms*.

[12] Oladeji O. (2016). The characteristics and roles of medicinal plants: some important medicinal plants in Nigeria. *Indian Journal of Natural Products*.

[13] Jamshidi-Kia F., Lorigooini Z., Amini-Khoei H. (2018). Medicinal plants: past history and future perspective. *Journal of Herbmed Pharmacology*.

[14] Namita P., R Mukesh R. (2012). *Kigelia pinnata*: a review. *Research Journal of Pharmacy and Technology*.

[15] Ahmed M. N. Pharmacological importance of medicinal plants. *Journal of Molecular Pharmaceuticals and Regulatory Affairs*.

[16] Assis F. V. D., Siqueira F. L., Goncalves I. E. (2018). Antibacterial activity of Lamiaceae plant extracts in clinical isolates of multidrug-resistant bacteria. *Anais da Academia Brasileira de Ciências*.

[17] Hostettmann K., Marston A., Ndjoko K., Wolfender J.-L. (2000). The potential of African plants as a source of drugs. *Current Organic Chemistry*.

[18] Moyo B., Mukanganyama S. (2015). Antiproliferative activity of *T. welwitschii* extract on Jurkat T cells *in vitro*. *BioMed Research International*.

[19] De Morais Lima G. R., De Sales I. R. P., Caldas Filho M. R. D. (2012). Bioactivities of the genus Combretum (combretaceae): a review. *Molecules*.

[20] Mangoyi R., Mafukidze W., Marobela K., Mukanganyama S. (2012). Antifungal activities and preliminary phytochemical investigation of Combretum species from Zimbabwe. *Journal of Microbial and Biochemical Technology*.

[21] Chigora P., Masocha R., Mutenheri F. (2007). The role of indigenous medicinal knowledge (IMK) in the treatment of ailments in rural Zimbabwe: the case of Mutirikwi communal lands. *Journal of Sustainable Development in Africa*.

[22] Masoko P., Picard J., Eloff J. N. (2007). The antifungal activity of twenty-four southern African Combretum species (Combretaceae). *South African Journal of Botany*.

[23] Obakiro S. B., Kiprop A., Kowino I. (2020). Ethnobotany, ethnopharmacology, and phytochemistry of traditional medicinal plants used in the management of symptoms of tuberculosis in East Africa: a systematic review. *Tropical Medicine and Health*.

[24] Maroyi A. (2013). Traditional use of medicinal plants in south-central Zimbabwe: review and perspectives. *Journal of Ethnobiology and Ethnomedicine*.

[25] Abubakar A., Haque M. (2020). Preparation of medicinal plants: basic extraction and fractionation procedures for experimental purposes. *Journal of Pharmacy and BioAllied Sciences*.

[26] Ngouana V., Zeuko’o Menkem E., Youmbi D. Y., Yimgang L. V., Toghueo R. M. K., Boyom F. F. (2021). Serial exhaustive extraction revealed antimicrobial and antioxidant properties of *Platycerium stemaria* (Beauv) Desv. *BioMed Research International*.

[27] Hamidian M., Blasco L., Tillman L. N., To J., Tomas M., Myers G. S. A. (2020). Analysis of complete genome sequence of *Acinetobacter baumannii* strain ATCC 19606 reveals novel mobile genetic elements and novel prophage. *Microorganisms*.

[28] Zhu Y., Lu J., Zhao J. (2020). Complete genome sequence and genome-scale metabolic modelling of *Acinetobacter baumannii* type strain ATCC 19606. *International Journal of Medical Microbiology*.

[29] Eucast (2002). Determination of minimum inhibitory concentrations (MICs) of antibacterial agents by broth dilution. *Clinical Microbiology and Infectious Diseases*.

[30] Eloff J. N. (1999). The antibacterial activity of 27 southern African members of the Combretaceae. *South African Journal of Science*.

[31] Du W., Sun C., Liang Z., Han Y., Yu J. (2012). Antibacterial activity of hypocrellin A against *Staphylococcus aureus*. *World Journal of Microbiology and Biotechnology*.

[32] El-Nakeeb M. A., Abou-Shleib H. M., Khalil A. M., Omar H. G., El-Halfawy O. M. (2011). Membrane permeability alteration of some bacterial clinical isolates by selected antihistaminics. *Brazilian Journal of Microbiology*.

[33] Stiefel P., Schmidt-Emrich S., Maniura-Weber K., Ren Q. (2015). Critical aspects of using bacterial cell viability assays with the fluorophores SYTO9 and propidium iodide. *BMC Microbiology*.

[34] O’Toole G. A. (2011). Microtiter dish biofilm formation assay. *Journal of Visualized Experiments: JOVE*.

[35] Malagoli D. (2007). A full-length protocol to test haemolytic activity of paly toxin on human erythrocytes. *Invertebrate Survival Journal*.

[36] Ray A., Dittel B. N. (2010). Isolation of mouse peritoneal cavity cells. *Journal of Visualized Experiments: JOVE*.

[37] Mombeshora M., Mukanganyama S. (2019). Antibacterial activities, proposed mode of action and cytotoxicity of leaf extracts from Triumfetta welwitschii against *Pseudomonas aeruginosa*. *BMC Complementary and Alternative Medicine*.

[38] Kundishora A., Sithole S., Mukanganyama S. (2020). Determination of the cytotoxic effect of different leaf extracts from *Parinari curatellifolia* (Chrysobalanaceae). *Journal of Toxicology*.

[39] Ghasemi M., Turnbull T., Sebastian S., Kempson I. (2021). The MTT assay: utility, limitations, pitfalls, and interpretation in bulk and single-cell analysis. *International Journal of Molecular Sciences*.

[40] Khameneh B., Iranshahy M., Soheili V., Fazly Bazzaz B. S. (2019). Review on plant antimicrobials: a mechanistic viewpoint. *Antimicrobial Resistance and Infection Control*.

[41] Wu H., Chen H., Zhang J. (2022). The anti-multidrug-resistant *Acinetobacter baumannii* study on 1,3-diamino-7H-pyrrolo [3, 2-f] quinazoline compounds. *Molecules*.

[42] Atef N. M., Shanab S. M., Negm S. I., Abbas Y. A. (2019). Evaluation of antimicrobial activity of some plant extracts against antibiotic susceptible and resistant bacterial strains causing wound infection. *Bulletin of the National Research Centre*.

[43] Friday A., Otalu O., Ukwubile C. A., Onoruoyiza O. V., Garba E. G. B. (2020). Use of medicinal plants as antimicrobial agents: a review of the Successes Story. *International Journal of Medicinal Plants and Natural Products (IJMPNP)*.

[44] Yusnawan E. (2013). The effectiveness of polar and non-polar fractions of *Ageratum conyzoides l.* to control peanut rust disease and phytochemical screenings of secondary metabolites. *Jurnal Hama dan Penyakit Tumbuhan Tropika*.

[45] Chaves J. O., de Souza M. C., da Silva L. C. (2020). Extraction of flavonoids from natural sources using modern techniques. *Frontiers of Chemistry*.

[46] McGaw L. J., Rabe T., Sparg S. G., Jäger A. K., Eloff J. N., Van Staden J. (2001). An investigation on the biological activity of Combretum species. *Journal of Ethnopharmacology*.

[47] Intorasoot A., Chornchoem P., Sookkhee S., Intorasoot S. (2017). Bactericidal activity of herbal volatile oil extracts against multidrug-resistant *Acinetobacter baumannii*. *Journal of Intercultural Ethnopharmacology*.

[48] Miyasaki Y., Rabenstein J. D., Rhea J. (2013). Isolation and characterization of antimicrobial compounds in plant extracts against multidrug-resistant *Acinetobacter baumannii*. *PLoS One*.

[49] Silén H., Salih E. Y. A., Mgbeahuruike E. E., Fyhrqvist P. (2023). Ethnopharmacology, antimicrobial potency, and phytochemistry of african *Combretum* and *pteleopsis* species (combretaceae): a review. *Antibiotics*.

[50] Mawire P., Mozirandi W., Heydenreich M., Chi G. F., Mukanganyama S. (2021). Isolation and antimicrobial activities of phytochemicals from *Parinari curatellifolia* chrysobalanaceae. *Advances in Pharmacological and Pharmaceutical Sciences*.

[51] Machingauta A., Stevens M. Y., Fru C. G., Sithole S., Yeboah S., Mukanganyama S. (2022). Evaluation of the antiproliferative effect of *β*-sitosterol isolated from *Combretum platypetalum* Welw. ex M.A. Lawson (Combretaceae) on Jurkat-T cells and protection by glutathione. *Advances in Traditional Medicine*.

[52] Musini A., Giri A. (2019). Investigation of mode of action of Anti-bacterial activity of *Salacia oblonga* extract against drug resistant pathogen. *Brazilian Archives of Biology and Technology*.

[53] Hao K., Xu B., Zhang G. (2021). Antibacterial activity and mechanism of *Litsea cubeba L*. Essential oil against *Acinetobacter baumannii*. *Natural Product Communications*.

[54] Zhang L., Xu S. G., Liang W. (2015). Antibacterial activity and mode of action of *Mentha arvensis* ethanol extract against multidrug-resistant *Acinetobacter baumannii*. *Tropical Journal of Pharmaceutical Research*.

[55] Arokiyaraj S., Bharanidharan R., Agastian P., Shin H. (2018). Chemical composition, antioxidant activity and antibacterial mechanism of action from *Marsilea minuta* leaf hexane: methanol extract. *Chemistry Central Journal*.

[56] Pakharukova N., Tuittila M., Paavilainen S. (2018). Structural basis for *Acinetobacter baumannii* biofilm formation. *Proceedings of the National Academy of Sciences*.

[57] Raja Yahya M. F. Z. (2020). Anti-biofilm potential and mode of action of malaysian plant species: a review. *Science Letter*.

[58] Gharbani P., Jam N., Doshmanfekan H., Mehrizad A. (2023). Optimization of synergic antibacterial activity of *Punica granatum* L. and *Areca nut* (P.G.L.A.N) extracts through response surface methodology. *Scientific Reports*.

[59] Chipenzi T., Baloyi G., Mudondo T., Sithole S., Fru Chi G., Mukanganyama S. (2020). An evaluation of the antibacterial properties of tormentic acid congener and extracts from *Callistemon viminalis* on selected ESKAPE pathogens and effects on biofilm formation. *Advances in Pharmacological and Pharmaceutical Sciences*.

[60] Lu L., Hu W., Tian Z. (2019). Developing natural products as potential anti-biofilm agents. *Chinese Medicine*.

[61] Sharma D., Misba L., Khan A. U. (2019). Antibiotics versus biofilm: an emerging battleground in microbial communities. *Antimicrobial Resistance and Infection Control*.

[62] Thouri A., Chahdoura H., Arem A. E., Hichri A. O., Hassin R. B., Achour L. (2017). Effect of solvent extraction on phytochemical components and biological activities of Tunisian date seeds (var. Korkobbi and Arechti). *BMC Complementary and Alternative Medicine*.

[63] Sánchez E., Rivas Morales C., Castillo S., Leos-Rivas C., García-Becerra L., Ortiz Martínez D. M. (2016). Antibacterial and antibiofilm activity of methanolic plant extracts against nosocomial microorganisms. *Evidence-based Complementary and Alternative Medicine*.

[64] Karunanidhi A., Ghaznavi-Rad E., Hamat R. A. (2018). Antibacterial and antibiofilm activities of nonpolar extracts of *Allium stipitatum* regel. against multidrug-resistant bacteria. *BioMed Research International*.

[65] Roy R., Tiwari M., Donelli G., Tiwari V. (2018). Strategies for combating bacterial biofilms: a focus on anti-biofilm agents and their mechanisms of action. *Virulence*.

[66] Jam N., Hajimohammadi R., Gharbani P., Mehrizad A. (2022). Antibacterial activity of *Punica granatum L*. and *Areca nut* (P.A) combined extracts against some food born pathogenic bacteria. *Saudi Journal of Biological Sciences*.

[67] Kalegari M., Miguel M. D., Dias J. D. F. G. (2011). Phytochemical constituents and preliminary toxicity evaluation of leaves from *Rourea induta Planch*. (Connaraceae). *Brazilian Journal of Pharmaceutical Sciences*.

[68] Falya Y., Sumiwi S. A., Jutti Levita J. (2020). Mini Review: toxicity study of plant extracts. *IOSR-JPBS*.

[69] Ghosh T. M., Biswas K., Chatterjee S., Roy P. (2018). *In-vitro* study on the hemolytic activity of different extracts of Indian medicinal plant Croton bonplandianum with phytochemical estimation: a new era in drug development. *Journal of Drug Delivery and Therapeutics*.

[70] Whitehead R. D., Mei Z., Mapango C., Jefferds M. E. D., Jefferds M. E. D. (2019). Methods and analyzers for hemoglobin measurement in clinical laboratories and field settings. *Annals of the New York Academy of Sciences*.

[71] International Organization for Standardization *Biological Evaluation of Medical Devices-Part 5 Tests for in Vitro Cytotoxicity*.

[72] Adegbaju O. D., Otunola G. A., Afolayan A. J. (2020). Anti-inflammatory and cytotoxic evaluation of extracts from the flowering stage of Celosia argentea. *BMC Complementary Medicine and Therapies*.

[73] Liu T., Liu F., Peng L. W., Chang L., Jiang Y. M. (2018). The peritoneal macrophages in inflammatory diseases and abdominal cancers. *Oncology Research Featuring Preclinical and Clinical Cancer Therapeutics*.

[74] Mapfunde S., Sithole S., Mukanganyama S. (2016). *In vitro* toxicity determination of antifungal constituents from *Combretum zeyheri*. *BMC Complementary and Alternative Medicine*.

[75] Arteaga F., Llbn M. P. V., Petricevich V. L. (2015). Preliminary studies of the immunomodulatory effect of the *Bougainvillea xbuttiana* extract in a mouse model. *Evidence-based Complementary and Alternative Medicine*.

[76] Ghonime M., Emara M., Shawky R., Soliman H., El-Domany R., Abdelaziz A. (2015). Immunomodulation of RAW 264.7 murine macrophage functions and antioxidant activities of 11 plant extracts. *Immunological Investigations*.

